# Codeine plus acetaminophen improve sleep quality, daily activity level, and food intake in the early postoperative period after photorefractive keratectomy: a secondary analysis

**DOI:** 10.5935/0004-2749.20210008

**Published:** 2025-02-02

**Authors:** Vinicius B. P. Pereira, André A. M. Torriceli, Renato Garcia, Samir J. Bechara, Milton R. Alves

**Affiliations:** 1 Departamento de Oftalmologia, Faculdade de Medicina, Universidade de São Paulo, São Paulo, SP, Brazil

**Keywords:** Codeine, Photorefractive keratectomy, McGill Pain Questionnaire, Pain, Acetaminophen, Sleep, Activities of daily living, Codeina, Ceratectomia fotorrefrativa, McGill Pain Questionnaire, Dor, Acetaminofen, Sono, Atividades cotidianas

## Abstract

**Purpose:**

To determine whether codeine plus acetaminophen after photorefractive keratectomy (PRK) have beneficial effects on sleep quality, activity levels, and food intake, beyond their effect of pain relief.

**Methods:**

We enrolled 40 patients (80 eyes) in this randomized, double-blind, paired-eye, placebo-controlled, add-on trial. Each eye was treated 2 weeks apart, and the patients were randomly allocated to receive either the placebo or the intervention (30 mg codeine and 500 mg acetaminophen) (4 times a day for 4 days). Outcomes were sleep quality, daily activity level, and food intake within 24-72 h post-photorefractive keratectomy, as measured by the McGill Pain Questionnaire.

**Results:**

Sleep quality and daily activity level were inversely associated with pain scores within the first 48 h post-photorefractive keratectomy. During the intervention, patients were significantly more likely to score their sleep quality as good at 24 h (relative risk=2.5; 95% confidence interval 1.48-4.21, p<0.001) and 48 h compared to during placebo (relative risk=1.37; 95% confidence interval: 1.03-1.84, p=0.023). The probability of reporting good daily activity level at 24 and 72 hours post-photorefractive keratectomy was three times higher when patients received the intervention compared to the placebo (relative risk=3.0; 95% confidence interval: 1.49-6.15, p=0.006 and relative risk=1.31; 95% confidence interval: 1.02-1.67, p=0.021, respectively). No difference was observed in food intake.

**Conclusion:**

The oral combination of codeine and acetaminophen significantly improves sleep quality and daily activity level within the first 24-72 h post-photorefractive keratectomy compared to a placebo.

**ClinicalTrials.gov number:** NCT02625753

## Abstract (pt)

**Objetivo:**

Determinar se codeína (30 mg) mais paracetamol (500 mg) após ceratectomia fotorrefrativa fornece efeitos benéficos sobre a qualidade do sono, níveis de atividade e ingestão de alimentos além de seu efeito analgésico.

**Métodos:**

Quarenta pacientes (80 olhos) foram incluídos neste estudo randomizado, duplo-cego, pareado, placebo-controlado*, add-on.* Cada olho foi tratado com 2 semanas de intervalo, sendo aleatoriamente alocado para placebo ou intervenção (4x/ dia durante 4 dias). Os resultados incluíram a qualidade do sono, atividade diária e ingestão de alimentos dentro de 24-72 horas de pós-operatório, conforme medido pelo McGill Pain Questionnaire.

**Resultados:**

A qualidade do sono e os níveis de atividade foram inversamente associados aos escores de dor nas primeiras 48 horas após o ceratectomia fotorrefrativa. Durante a intervenção, os pacientes foram significativamente mais propensos a classificar seu sono como bom em 24 horas (risco relativo=2,5, intervalo de confiança de 95%: 1,48-4,21, p<0,001) e 48 horas comparado ao placebo (risco relativo=1,37, intervalo de confiança de 95%: 1,03-1,84, p=0,023). A probabilidade de relatar bons níveis de atividade em 24 e 72 horas após ceratectomia fotorrefrativa também foi significativamente maior durante a intervenção em comparação com placebo (risco relativo=3,0, intervalo de confiança de 95%: 1,49-6,15, p=0,006 e risco relativo=1,31, intervalo de confiança de 95%: 1,02 -1,67, p=0,021, respectivamente). Nenhuma diferença foi observada entre a intervenção e placebo em relação à alimentação oral.

**Conclusão:**

A combinação de codeína e paracetamol melhorou significativamente a qualidade do sono e atividades diárias nas primeiras 24-72 horas após o ceratectomia fotorrefrativa em comparação com placebo.

## INTRODUCTION

Excimer laser photorefractive keratectomy (PRK) is a surgical procedure commonly performed for correcting mild-to-moderate refractive errors^(1,2)^. It is a preferred approach to treating special cases, such as a thin cornea, moderate dry eye, and subtle topographic irregularities, and patients who underwent previous eye surgeries^(3,4)^. However, despite its effectiveness and safety, PRK usually involves a high level of postoperative pain and discomfort^(5)^, which not only adversely affects the patient’s overall satisfaction with the procedure but also reduces his or her willingness to undergo the procedure again^(6,7)^. Therefore, improvement of patient care during the immediate PRK postoperative period has become a major clinical challenge for ophthalmologists^(8-12)^.

We recently demonstrated that adding an oral combination of codeine and acetaminophen to a standardized postoperative pain regimen is an effective and safe therapeutic strategy to alleviate post-PRK pain^(8)^. This approach provides significantly greater pain relief compared to a placebo without affecting corneal wound healing or having serious adverse effects. However, whether this therapeutic strategy also leads to improvement in subjective indexes of health and well-being in the early PRK postoperative period is unclear. It is conceivable that improvements in personal, social, and daily activity-related contexts post-PRK might potentially lead to better patient satisfaction and clinical outcomes, ultimately having profound implications for PRK’s popularity and acceptability^(7)^. Surprisingly, to the best of our knowledge, no clinical trial has specifically performed a multidimensional analysis of how a pain management strategy affects health-related quality of life outcomes post-PRK.

In light of the limited data on the benefits of systemic opioids on improving patient care post-PRK, we performed a post-hoc secondary analysis based on our previous randomized clinical trial in order assess whether, compared to a placebo, the oral combination of codeine and acetaminophen provides beneficial effects on sleep quality, daily activity level, and food intake in patients post-PRK. This study will provide a more detailed assessment of the feasibility of using this therapeutic strategy in clinical practice.

## METHODS

### Study design, participants, and outcomes

The trial design, eligibility criteria, outcomes, and postoperative pain protocol have been reported previously^(8)^. Briefly, we performed a randomized, doubleblind, paired-eye, placebo-controlled, add-on trial (ClinicalTrials. gov number: NCT02625753). We assessed the efficacy and safety of the oral combination of codeine and acetaminophen compared to a matching placebo for pain reduction post-PRK. Between November 2014 and June 2015, 228 patients were admitted to the Hospital das Clínicas, Universidade de São Paulo, São Paulo, Brazil, between November 2014 and June 2015 for PRK. We excluded pregnant or lactating women and patients with hypersensitivity or allergy to oral medications, active allergic disease, inflammatory, or infectious conditions, a history of ocular disease or trauma, bestcorrected visual acuity ≤20/25, autoimmune diseases or immunosuppression, or type I/II diabetes. Inclusion criteria were as follows: patients scheduled for myopic excimer laser PRK, age ≥20 years, eyes with a spherical component between -1.00 and -5.00 diopters (D) with or without astigmatism, cylindrical component ≤1.5 D, spherical anisometropia ≤0.75 D, cylindrical anisometropia ≤0.5 D, and documented refractive stability over the previous 12 months. Therefore, 41 patients (82 eyes) met all eligibility criteria. Of them, 1 patient who had recurrent vomiting after the first surgery and was found to have celiac disease was excluded. Thus, a total of 40 patients (80 eyes) participated in the trial, of which 27 (67%) were women and most were white (57%).

The original primary outcome was a difference in pain levels between codeine+acetaminophen-treated eyes and placebo-treated eyes, as measured on a 0-10 pain visual analog scale (VAS) obtained 24 h post-PRK. A detailed list of all secondary outcomes is available in our previous publication.

The trial was approved by the local ethics committee and adhered to the Declaration of Helsinki. All participants provided written informed consent.

### PRK, procedures, and drug administration

All surgeries were performed by the same surgeon. The unit of analysis was the eye. All patients underwent bilateral PRK, but each eye was treated 2 weeks apart. Patients were randomly administered either the intervention or the placebo in the form of indistinguishable capsules containing either 30 mg of codeine and 500 mg or acetaminophen or the placebo (1:1 ratio). Blinding and allocation concealment were maintained by the use of identical, coded medication bottles prepared by an independent pharmacist. Randomization was controlled by the dispensing pharmacy that dispensed either the intervention or the placebo using computer-generated random numbers. Treatment was given orally 4 times a day for 4 days post-PRK.

In addition, all patients received standard care as per the hospital protocol: a regimen of 200 mg of celecoxib twice a day for 4 days started 1 h before PRK, and a drop each of 0.5% moxifloxacin and 0.1% dexamethasone immediately post-PRK but before receiving an Acuvue II therapeutic contact lens (Johnson & Johnson, New Brunswick, NJ, USA). Patients were instructed to use 0.5% moxifloxacin and 0.1% dexamethasone ophthalmic drops every 4 h for 7 days, 1 mg/mL of nepafenac ophthalmic suspension every 6 h for 3 days, and artificial tears, as needed, without any concurrent medications for 72 h post-PRK. One week post-PRK, patients received 1 mg/mL of fluorometholone eye drops every 8 h for 7 days, with increasing dose intervals: every 12 h in week 3 and once per day in week 4.

### Outcomes in secondary analysis

Findings for patient-reported outcomes were reported on the basis of three domains of the McGill Pain Question naire: sleep quality, daily activity level, and food intake. Sleep quality was measured on a 3-point Likert scale (good, fitful, or can’t sleep). Both daily activity level and food intake were operationalized on a 4-point Likert scale (good, some, little, none), as previously described^(13)^.

### Statistical analysis

Data were presented as mean (95% confidence intervals [CIs]) or counts (percentage). To test the effects of the intervention compared to the placebo, we constructed multinomial logistic and multiple linear regression models (adjusted for age, gender, and race). These models explicitly incorporated the paired-eye design using a robust estimator of the variance, which took into account the correlation between pairs of eyes. Relative risks [RRs] and 95% CIs were calculated, as described previously^(14)^. All analyses were performed using Stata 14 (Stata Corp, College Station, TX, USA). P was two-tailed, and p<0.05 was considered statistically significant.

## RESULTS

A description of the patient population has been reported previously^(8)^ and will be briefly summarized here. The mean age of the patients was 30 years (min-max=22-52 years). The mean spherical equivalent was -2.18 (0.66) in the right eye and -2.16 (0.63) in the left eye.

### Sleep quality

Post-PRK sleep quality assessment revealed an inverse association between pain scores and sleep quality within the first 24 h post-PRK (Table 1); the higher the pain levels at 24 h, the lower the sleep quality (p for trend=0.008). However, we did not observe robust and statistically significant correlations between pain levels and sleep quality at 48 and 72 h post-PRK (Table 1). Remarkably, at 24 and 48 h post-PRK, patients receiving the intervention were significantly more likely to score their sleep as good compared to patients receiving the placebo (58% vs. 25%; p<0.001 and 82% vs. 60%; p=0.02, respectively) (Table 2).

**Table 1:** Association between subjective indexes of health, personal and social contexts, and pain levels

Time point		Category	Eyes (n=80)	Pain levels (cm), mean (95% CI)	P (overall)	P (trend)	β in cm (95% CI)	P (coefficient)	
Sleep quality 24h		Good	33 (41)	4.60 (3.85,5.35)	0.003	0.008	Reference		
		Fitful	38 (47)	6.52 (5.74,7.30)			1.92 (0.85,2.98)	0.001	
		Can’t sleep	9 (11)	6.29 (4.67,7.91)			1.69 (-0.11,3.48)	0.06	
48h		Good	57 (71)	2.19 (1.71,2.67)	0.37	0.37	Reference		
		Fitful	23 (29)	2.61 (1.90,3.31)			0.41 (-0.51,1.34)	0.37	
		Can’t sleep	0	-			-	-	
72h		Good	75 (93)	0.53 (0.30,0.76)	0.85	0.63	Reference		
		Fitful	4 (5)	0.63 (0.00,1.60)			0.10 (0.00,1.13)	0.85	
		Can’t sleep	1 (1)	0			-	-	
	Daily activities								
	24h	Good	10 (13)	4.48 (2.99,5.96)	0.16	0.04	Reference		
		Some	6 (7)	5.18 (4.22,6.15)			0.70 (-0.75,2.16)	0.33	
		Little	25 (31)	5.25 (4.43,6.07)			0.77 (-0.94,2.49)	0.37	
		None	39 (49)	6.38 (5.39,7.37)			1.90 (0.05,3.75)	0.04	
	48h	Good	24 (30)	2.01 (1.25,2.77)	0.05	0.018	Reference		
		Some	24 (30)	2.01 (1.35,2.67)			0 (-1.03,1.03)	0.99	
		Little	26 (33)	2.49 (1.98,3.01)			0.48 (-0.50,1.46)	0.32	
		None	6 (7)	3.93 (2.65,5.21)			1.92 (0.49,3.34)	0.01	
	72h	Good	53 (66)	0.46 (0.19,0.72)	0.56	0.58	Reference		
		Some	15 (19)	0.77 (0.30,1.25)			0.32 (-0.22,0.85)	0.24	
		Little	11 (14)	0.56 (0.06,1.06)			0.11 (-0.48,0.69)	0.72	
		None	1 (1)	0.14 (0.00,0.82)			-0.31 (-0.93,0.30)	0.31	
Oral feeding 24h		Good	40 (50)	5.43 (4.56,6.30)	0.30	0.18	Reference		
		Some	14 (17)	5.39 (4.03,6.75)			-0.04 (-1.79,1.70)	0.96	
		Little	26 (33)	6.28 (5.31,7.26)			0.85 (-0.38,2.09)	0.17	
		None	0	-			-	-	
48h		Good	61 (76)	2.16 (1.72,2.60)	0.26	0.14	Reference		
		Some	13 (16)	2.91 (2.12,3.70)			0.75 (-0.18,1.68)	0.11	
		Little	6 (7)	2.58 (1.43,3.72)			0.42 (-0.82,1.65)	0.50	
		None	0	-					
72h		Good	73 (91)	0.55 (0.32,0.78)	0.09	0.64	Reference		
		Some	5 (6)	0.06 (0.00,0.45)			-0.49 (-0.97,-0.02)	0.04	
		Little	2 (3)	0.65 (0.00,2.26)			0.10 (-1.54,1.73)	0.90	
		None	0	-			-	-	

Pain levels are from 0 to 10 VAS (cm).

β is the mean difference comparing a specific category to the reference group (in cm).

All results are adjusted for race, age, gender, and treatment (1=codeine with acetaminophen; 0=placebo) and take into account the correlation between pairs of eyes. *P* (overall): ANOVA-like test that examines whether there is any difference between groups. *P* (trend): tests the linear trend in the mean over categories (sleep quality: 0=good; 1=fitful; 2=can’t sleep; daily activities and food intake: 0=good; 1=some; 2=little; 3=none). *P* (coefficient): examines whether specific categories differ from the reference category.

CI= confidence intervals; VAS= visual analog scale; ANOVA= analysis of variance.

**Table 2:** Effect of the intervention (codeine and acetaminophen) compared to the placebo on subjective indexes of health and personal and social contexts

Time point	Category	Intervention		Placebo		RR (95% Cl)	*P*
		N	%	N	%		
Sleep quality 24h	Good	23	58	10	25	2.50(1.48-4.21)	<0.001
	Fitful	16	40	22	55	0.63 (0.46-0.87)	0.002
	Can’t sleep	1	2	8	20	0.25 (0.13-0.51)	0.009
48h	Good	33	82	24	60	1.37(1.03-1.84)	0.02
	Fitful	7	19	16	40	0.44(0.20-0.95)	0.02
	Can’t sleep	0	0	0	0	NE	NE
72h	Good	40	100	35	88	1.05 (0.93-1.18)	0.44
	Fitful	0	0	4	10	0.25 (0.03-2.28)	0.29
	Can’t sleep	0	0	1	2	NE	NE
Daily activities 24h	Good	6	15	4	10	3.03 (1.49-6.15)	0.006
	Some	5	12	1	2	2.32 (1.29-4.14)	0.005
	Little	16	40	9	23	1.44 (1.04-1.99)	0.02
	None	13	33	26	65	0.54 (0.36-0.81)	0.001
48h	Good	15	38	9	22	1.66 (0.86-3.22)	0.10
	Some	15	38	9	22	1.66 (0.85-3.27)	0.11
	Little	8	20	18	45	0.44 (0.23-0.88)	0.006
	None	2	5	4	10	0.50 (0.12-2.03)	0.32
72h	Good	30	75	23	58	1.31 (1.02-1.67)	0.02
	Some	6	15	9	23	0.65 (0.42-1.01)	0.06
	Little	4	10	7	17	0.50 (0.26-0.94)	0.02
	None	0	0	1	2	0.44 (0.21-0.91)	0.03
Oral feeding 24h	Good	23	58	17	42	1.35 (0.91-2.00)	0.13
	Some	10	25	4	10	2.50 (0.85-7.32)	0.09
	Little	7	17	19	48	0.37 (0.17-0.77)	0.008
	None	0	0	0	0	NE	NE
48h	Good	32	80	29	73	1.13 (0.91-1.38)	0.25
	Some	7	18	6	15	0.71 (0.39-1.29)	0.26
	Little	1	2	5	12	0.62 (0.27-1.42)	0.26
	None	0	0	0	0	NE	NE
72h	Good	39	98	34	85	1.15 (1.01-1.30)	0.03
	Some	1	2	4	10	0.17 (0.03-0.98)	0.05
	Little	0	0	2	5	0.14 (0.02-0.84)	0.03
	None	0	0	0	0	NE	NE

CI= confidence interval; RR= relative risk; NE= not estimated (due to sparse data).

### Daily activity level

Analysis of the daily activity level post-PRK indicated that higher pain levels are associated with less activity within the first 48 h post-PRK (p for trend=0.039 and 0.018 at 24 and 48 h, respectively) (Table 1). The probability of reporting good daily activity levels at 48 h post-PRK was three times higher when patients received the intervention compared to the placebo (RR=3.0; 95% CI=1.5-6.1; p=0.006). In addition, although at 48 and 72 h, the effect sizes were smaller, the results were qualitatively analogous (RR=1.6; 95% CI=0.9-3.2; p=0.10 and RR=1.31; 95% CI=1.0-1.7; p=0.021, respectively) (Table 2).

### Food Intake

The patients’ food intake was not associated with pain levels post-PRK (Table 1). When questioned about their food intake post-PRK, all patients reported adequate food intake throughout the 72 h period post-PRK (Table 2).

## DISCUSSION

### Main findings

The addition of an oral combination of codeine and acetaminophen to the post-PRK pain control protocol might improve the patient’s quality of life in the early recovery period post-PRK (24-48 h). Together with its efficacy in alleviating acute postoperative pain^(8)^, this therapeutic strategy might lead to a higher postoperative quality of life and is likely to positively affect the patient’s perception of PRK.

### Comparison with previous investigations

Postoperative pain management in PRK has been explored by many researchers in recent years^(5,8-12,15,16)^. However, subjective indexes of health and well-being that take place outside the clinical encounter have been rarely investigated and are typically disregarded in clinical practice by ophthalmologists. Indeed, although the early postoperative period post-PRK should be evaluated as a multidimensional concept^(7)^, no studies have specifically addressed health-related quality of life outcomes post-PRK.

Even though many factors alter sleep architecture and sleep quality, pain is a major cause of postoperative sleep disturbance in several medical specialties^(17)^. Notably, sleep disturbance might adversely affect postoperative recovery. In fact, sleep disturbance, commonly experienced by patients in the early PRK postoperative period, is associated with compromised patient recovery, longer hospitalization^(18)^, and reduced quality of life in non-ophthalmologic surgeries^(19)^. In addition, even shortterm impaired sleep quality might modify the patient’s sensitivity to nociceptive stimuli^(20)^, increa sing pain perception^(21)^, which can exacerbate sleep disturbance. A previous observational study showed that poor sleep quality might increase the risk of dry eye^(22)^ and exacerbate or prolong highly prevalent transient ocular surface disease^(6,23,24)^. Therefore, therapeutic strategies, such as the combination of codeine and acetaminophen, capable of improving sleep, can optimize the early postoperative PRK recovery period and likely result in improved clinical outcomes and greater patient satisfaction.

We are not aware of any randomized trials that have compared the effects of pain reduction strategies on a patient’s activities post-PRK. However, the higher daily activity level during intervention, compared to the placebo, could be explained, at least in part, by decreased pain levels and better sleep quality. In fact, both pain levels and sleep quality strongly affects the patient’s daily activity level^(25,26)^.

### Limitations

This trial had a few limitations. First, the analysis was limited by a relatively low statistical power to detect small to moderate effects. Second, the Likertbased component of the McGill Pain Questionnaire does not capture the complex multidimensionality of the patient’s quality of life. Other health-related quality of life instruments, such as SF-12/36^(27)^, might evaluate a range of different health domains and are likely to be more suitable for further, more comprehensive investigations.

The oral combination of codeine and acetaminophen significantly improves sleep quality and the daily activity level within the first 24-72 hours post-PRK compared to a placebo, which reinforces the feasibility of this therapeutic strategy for use in post-PRK pain management. Our findings highlight the importance of both patientreported outcomes and patient-centered type of care post-PRK to increase the popularity and acceptability of this common laser vision correction procedure.
